# Modular E-Collar for Animal Telemetry: An Animal-Centered Design Proposal

**DOI:** 10.3390/s22010300

**Published:** 2021-12-31

**Authors:** Marta Siguín, Teresa Blanco, Federico Rossano, Roberto Casas

**Affiliations:** 1 Howlab (Human Openware Research Lab) Research Group, I3A (Aragon Institute of Engineering Research), University of Zaragoza, 50009 Zaragoza, Spain; msiguin@unizar.es (M.S.); tblanco@unizar.es (T.B.); 2 GeoSpatium Lab S.L., Carlos Marx 6, 50015 Zaragoza, Spain; 3 CCL (Comparative Cognition Lab), University of California, San Diego, CA 92093, USA; frossano@ucsd.edu

**Keywords:** wearables design, animal farming, animal-centered design, animal telemetry, modularity, smart collar, design contributions, additive manufacturing

## Abstract

Animal telemetry is a subject of great potential and scientific interest, but it shows design-dependent problems related to price, flexibility and customization, autonomy, integration of elements, and structural design. The objective of this paper is to provide solutions, from the application of design, to cover the niches that we discovered by reviewing the scientific literature and studying the market. The design process followed to achieve the objective involved a development based on methodologies and basic design approaches focused on the human experience and also that of the animal. We present a modular collar that distributes electronic components in several compartments, connected, and powered by batteries that are wirelessly recharged. Its manufacture is based on 3D printing, something that facilitates immediacy in adaptation and economic affordability. The modularity presented by the proposal allows for adapting the size of the modules to the components they house as well as selecting which specific modules are needed in a project. The homogeneous weight distribution is transferred to the comfort of the animal and allows for a better integration of the elements of the collar. This device substantially improves the current offer of telemetry devices for farming animals, thanks to an animal-centered design process.

## 1. Introduction

Telemetry combines the use of different sensors and wireless communications to perform physical and/or chemical measurements remotely. Applied to the study of animals, it allows for the acquisition of animal life data through a device placed on the animal that sends signals to a receptor [1]. In this way, different issues related to the individual and their environment can be monitored in a much less invasive way, without having to come into direct contact with them, except for the placement of the device.

Since the 1960s, radiotelemetry has been used as an instrument to track the position of animals and study their behavior [2,3,4]. In the last few years, the development of telemetry devices in animal studies has provided noteworthy advances in the direction of increasing the batteries’ lifetime, improving the precision and functionality of systems, miniaturizing devices, increasing the variety and novelty of data collected, and research in data processing as well as the use of eco-friendly materials and renewable energy sources [5,6,7,8,9,10].

Recently, small sensors from mobile and communication technologies and location systems have gradually been integrated in animal telemetry: accelerometers, magnetometers, cameras, temperature sensors, pressure sensors, etc. They have been combined and placed in global positioning system (GPS) collars, allowing for the study of ecological issues around migration, foraging behavior, physiological performance, habitat selection and social interaction, particularly, of medium and large terrestrial mammals [4,11].

Therefore, telemetry has become a very powerful tool in the study of animal life for the purposes of evolutionary, behavioral, and veterinary research; for the monitoring of animals in their environment; and for the conservation of fauna. It has brought greater efficiency and objectivity, and has allowed professionals to work with animals that would have been unthinkable to study in their habitat just a few years ago. In addition, the automation of animal monitoring ensures the continuity of data collection, which becomes an obstacle in extreme situations such as the one we currently experience due to *Coronavirus Disease 2019* (COVID-19) [12,13].

In the field of smart livestock, monitoring has led to the improvement in animal protection and welfare through the monitoring of their behavioral and physiological states, which represents a step toward the responsible production and consumption of animal materials. Monitoring allows (i) to improve the traceability of animal welfare; (ii) facilitate decision-making to which the farmer submits; and (iii) favor the management of the exploitation [6,9,13,14,15].

The application of telemetry in animals presents difficulties, mainly related to (i) weight distribution; (ii) autonomy; (iii) flexibility in design; and (iv) cost of the devices. These problems have become apparent from the analysis of three types of sources: scientific publications referring to the physical design of animal telemetry devices [16,17,18,19,20,21,22,23,24]; scientific publications referring to the evaluation of animal telemetry [13,25,26,27] as well as commercial solutions currently available [28,29,30,31,32,33,34,35,36].

Most of these problems are highly dependent on design. Design is a process capable of connecting technology with the real requirements of users, providing the market with products and services that respond to the diverse cultural and social context in which we live, which currently requires an indispensable technological adaptation. On one hand, a good design strategy brings innovation to the processes (i) of contextual and user research; (ii) detection of needs and definition of functionalities and requirements; (iii) of ideation and conceptualization; and (iv) finally evaluation. This can help to solve problems meshing the product, user, and environment as well as planning and formulating multidisciplinary strategies thanks to the holistic training of the designer, accustomed to working in a team and in various areas not related to their discipline [37]. On the other hand, design can also contribute to technical aspects of product development, defining it structurally and formally, and analyzing and making a good choice of materials and manufacturing processes.

This paper describes the design and evaluation of a low-cost telemetric device for the study of the behavior of medium and large mammals such as farm animals, which provides solutions to the problems detected in current devices. We hope to contribute both at a methodological level and in order to facilitate and extend the use of animal telemetry for the study of animals and their environment.

## 2. Materials and Methods

### 2.1. Design Concept 

**Figure 1:**
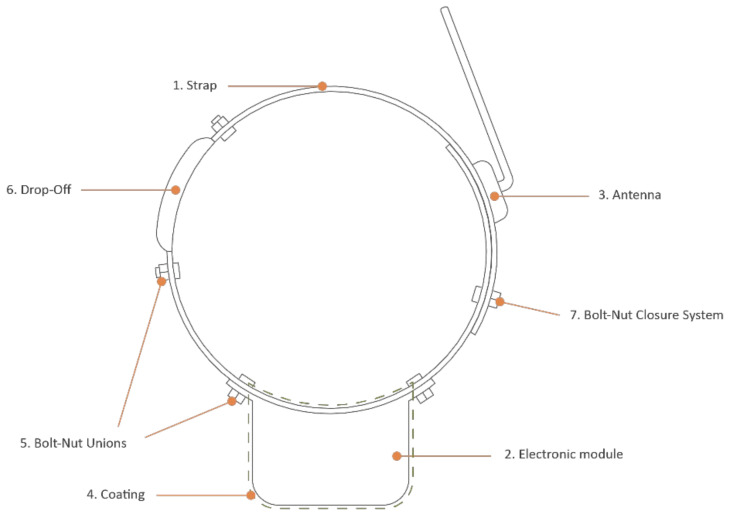
Main parts of a telemetry collar.

The structure of a typical telemetry collar is mainly composed of the following parts (Figure 1):

1. Strap: structural element on which the portability of the device is based;
2. Electronic module: envelope that contains the active part of the device inside. In general, collars have a single electronic module that is placed in the lower part of the animal’s neck, allowing the antenna to be correctly oriented thanks to the action of gravity;
3. Antenna: it is the component that allows the transmission of the information collected by the electronic device. The antenna can be external, a wire rope or a more sophisticated independent element such as the one in Figure 1, or internal, integrated into a printed circuit board (PCB);
4. Coating: sometimes electronic modules and/or antennae are covered with plastic materials to protect them;
5. Unions: the connection of the external elements with the strap is usually carried out by rivets or bolt–nut unions;
6. Drop-Off: this is a mechanism used in the field of wildlife to be able to recover the device without having to recapture the animal that carries it. These devices can be electronic or mechanical. The latter are based on the degradation of the material that composes them; when the material has degraded in the expected time, the collar falls off and can be recovered by scientists;
7. Closure system: the safest closures are made by means of two bolt–nut connections.

### 2.2. Design Methodology

**Figure 2:**
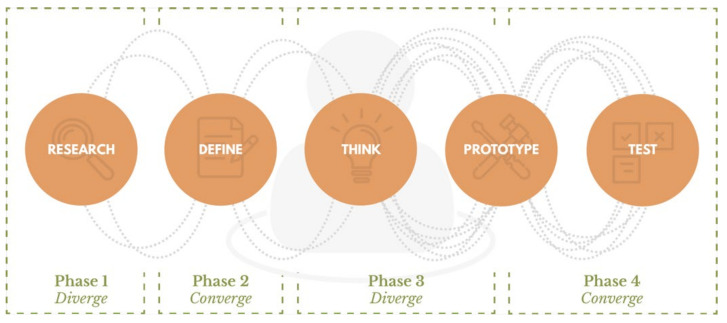
Methodological process of the investigation.

The design process followed for the development of this telematic device (Figure 2) was established based on basic design methodologies and approaches: (i) the Double Diamond of the British Design Council [38]; (ii) the ideology of People-Centered Design of IDEO [39]; (iii) the Design Thinking process of the D. School [40]; and (iv) of the design applied to IoT: Cosica [41,42]. These methodologies have been oriented to the experience of the animal and the human and to the design of wearables, and have been adapted to the context and ecosystem in which the project was developed.

A collaborative design process was carried out, in which the following had participated: designers, electronic engineers, and telecommunications engineers as well as veterinary experts, technical personnel who work in continuous contact with animals, behavioral researchers, and coordinators of animal centers.

#### 2.2.1. Phase 1–Research (Diverge)

The objective of this phase is to collect as much information as possible from users and the context, in order to subsequently define requirements based on their real needs. To achieve this, the following strategy is proposed (Table 1).

#### 2.2.2. Phase 2–Define (Converge)

After the divergence of the previous phase, where a large amount of information has been collected, in phase 2, it is intended to define the problem and divide it, in order to tackle it more easily. To do this, in the first place, the information collected is synthesized to highlight the most relevant, which helps to correctly focus the ideation process. This is done through *Clustering*, grouping the most important revelations in relation to the problems detected and the design requirements to be considered. Subsequently, designers, electronic engineers, and telecommunications engineers work together to propose and define technical solutions to the problems detected and to define the electronic components that the device will have in order to consider them in the design process. Finally, the defined problem is divided by presenting 12 design challenges that will guide the next creative stages.

The structural proposed challenges are:

- Challenge 1 (Body—Strap): Choice of materials and strap size;
- Challenge 2 (Body—Modules): How can a watertight and flexible union of modules be made?
- Challenge 3 (Body—Modules): How can a cover–body joint of the module be watertight and safe?
- Challenge 4 (Body—Modules): How can the union or registration between the modules and the strap be made?
- Challenge 5 (Body—Modules): What should the shape of the modules be so that they do not cause discomfort to the animal?
- Challenge 6 (Body—Cover): How should the modules be protected?
- Challenge 7 (Drop-Off): Adaptation and development of a Drop-Off system based on the degradation of latex tubes;
- Challenge 8 (Closure): Proposal of a rapid closing system; and
- Challenge 9 (Distribution): Proposal of a correct distribution of the elements along the strap.

These challenges must be carried out taking into account three transversal challenges:

- Challenge 10 (Environmental conditions): Design of a collar resistant to environmental conditions;
- Challenge 11 (Integration): Design of a compact collar whose parts are integrated; and
- Challenge 12 (Impact): Design a collar that has the least impact on the animal.

#### 2.2.3. Phase 3–Think and Prototype (Diverge)

**Figure 3:**
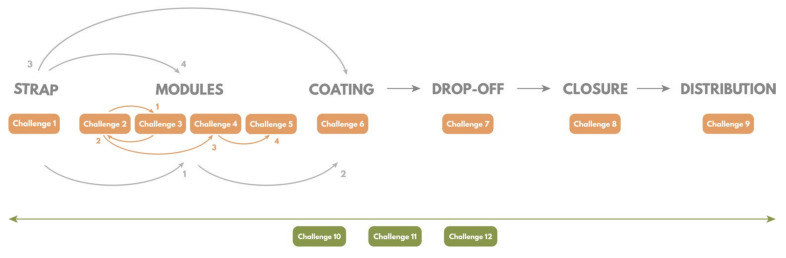
Iterative temporal development that was performed during the ideation process.

This phase aims to solve the challenges defined in the previous phase. The process carried out to achieve this phase has a highly iterative component (Figure 3). The system of challenges defined in the previous phase is followed to tackle the problems to be solved in a structured and defined way. As ideas are generated, they are prototyped and/or evaluated with users and the team to rule out options or to validate them.

#### 2.2.4. Phase 4–Prototype and Test (Converge)

In order to test the proposals: two final prototypes of the collar were made, where all the variations are represented, and a final evaluation was structured.

Evaluation is one of the key points of any process. The literature on the design of animal monitoring devices always structures its discourse taking into account a final evaluation that assesses the results of the use of the new proposed product. However, in most cases, these results focus only on technological deployment (autonomy, signal range, failed devices, etc.), and in the case of evaluating the physical design of the device, these studies serve few and very superficial objectives (for example, the device has been broken, has caused injuries to animals, or the mortality rate) [16,17,18,19,20,21,22,23,24]. In addition, these evaluations are merely quantitative, not giving value to the experience of the professionals.

Our complete evaluation of the design elements and of the overall design of the collar was carried out using a mixed methods approach [43,44,45].

In such a particular context as the one in which we find ourselves, the use of both quantitative and qualitative evaluation methods can help us (i) to complement the information when collecting data on dimensions that have only been evaluated by a single method (the adequacy of the force applied to close the collar or autonomy); (ii) to generate a more complete concept of the objectives through the combination (iterative methods with users have been combined with semi-structured interviews, so that the information obtained through the iteration has helped us to identify key points to deal in interviews); and (iii) and to refine the results by triangulating information on the same dimension (weight, ease of use).

##### Sources

The collar was evaluated through various sources:

- Experts in the Environment (EE): Managers of animal centers and workers, who act as potential clients and animal experts. They work with the animals and put the collar on them;
- Research Experts (RE): Behavioral researchers, also acting as potential clients and experts in animal interaction in a context of behavioral research;
- Engineers (E), who evaluate technical specifications of the collar in the laboratory; and
- Current Offer (CO), which allows the proposed collar to be evaluated against current designs.

##### Methods

- Laboratory Experiments (LE): Laboratory tests were carried out at different times in the process to evaluate technical issues such as tightness. Rapid prototyping techniques were also used to evaluate the physical designs of the parts and the distribution of weights;
- Iterative Methods with users (IM): Regular contact with experts was maintained. Through various methods such as meetings, open interviews, small product presentations, sending samples, etc., information was extracted on their opinions and judgments. These methods guided the design process and allowed us to detect elements that should be emphasized in future evaluations;
- Focus Group (FG): A focus group was held with four experts in behavioral science with extensive experience with animals. The objective of the focus group was to gain the opinions that research experts have in relation to the proposals and what they can contribute to their work;
- Real-Life Testing (RLT): The prototypes were evaluated with animals, which allowed the designers to observe how they relate to the morphometry of the animal. On the other hand, experts in the environment also observed the behavior of animals in relation to the collars. The collars were tested on sheep (rasa aragonesa and roya bilbilitana), goats (murciano granadina and mestiza de Florida), and horses (hispano-bretón) under the approval of the Ethical Committee of the University of Zaragoza (PI55/20, 28 October 2020);
- Semi-Structured Interviews (SSI): Semi-structured interviews were carried out with the experts in the environment to evaluate the alternatives reflected in the prototypes, which are detailed later; and
- Document Analysis (DA): To evaluate the proposals against the current panorama on animal telemetry, a table was compiled in which the characteristics of different collars on the market were compared.

Table 2 shows the objectives evaluated in relation to the sources and the methods used for their evaluation.

## 3. Results

As stated before, we aimed to solve four main problems detected in animal telemetry devices: weight distribution, autonomy, flexibility in design, and price. We proposed the design of a modular collar that distributes the electronic components in several compartments, connected and powered by rechargeable batteries. The manufacturing of the device was based on 3D printing.

**Figure 4:**
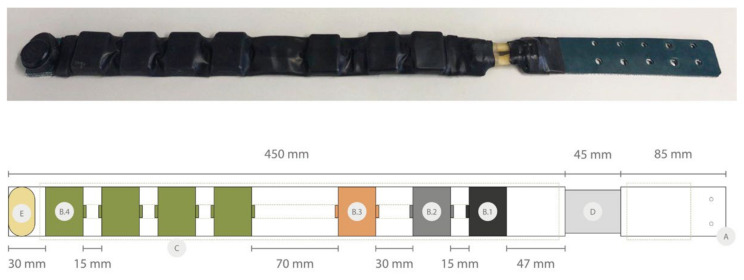
Bounded distribution of the elements of the collar (A Strap; B.1 GPS module; B.2 Communications module; B.3 Sensors module; B.4 Battery modules; C Coating; D Drop-Off; E Closure).

The distribution of the elements must bear in mind two main premises: (i) distributing the weight as evenly as possible along the collar so that the animal does not suffer; and (ii) that the antennae are always in the most convenient position of the collar to allow proper communications (e.g., GPS/satellite must be at upper position pointing to the sky). A low-level prototype was created to check and adjust the distribution of the elements, which was based on the balance of weights. The device needs at least six modules. However, to demonstrate the modularity and customization of the proposal, we decided to prototype a collar with seven modules, where four of them are batteries. This results in the composition depicted in Figure 4.

**Figure 5:**
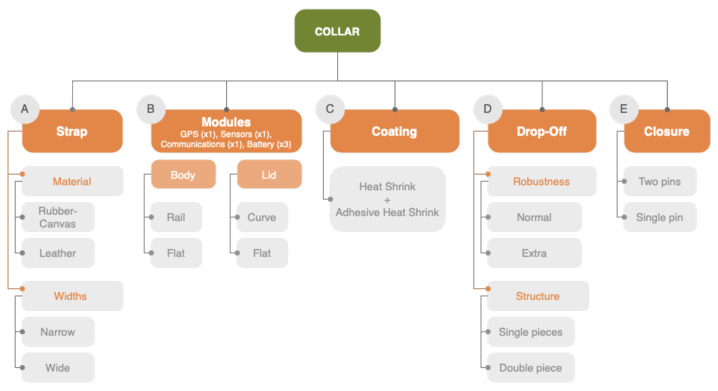
Block diagram of collar structure and design options.

As seen in Figure 5, the main structure of the collars can be divided into (A) strap; (B) modules, which are divided into body and lid; (C) coating; (D) drop-off; (E) and closure.

Several options are proposed for each of the structural elements in order to evaluate them (Figure 4).

### 3.1. Strap

The strap (Figure 4A) is the structural element on which the rest of the components are mounted. The choice of the material of the strap is a decision that revolves around its rigidity and its response to handling and weather conditions, since it will not come into contact with the animal’s skin because of the coating.

The use of two different materials (Figure 5A) was considered: a rubber–canvas composite material with several interleaved layers and natural leather. The leather strap was less rigid, more malleable, and adaptable to the movement of the animal, which can be more comfortable for the animal but also less resistant to pulling or biting, while the rubber–canvas strap was more rigid and helps define the shape of the collar, reversing the advantages and disadvantages compared to leather.

Various strap widths (Figure 5A) were also assessed, one equal to the height of the modules containing the electronics, so that the collar is more compact and is more protected from the action of animals; and another a little lower, with the idea of reducing the material to make it more flexible and comfortable for the animal.

### 3.2. Modules

#### 3.2.1. Shell

To favor the fractionation of the electronics, they were housed in independent but interconnected modules. These modules must be watertight in all of their joints and have a shape that is comfortable for the animal.

**Figure 6:**
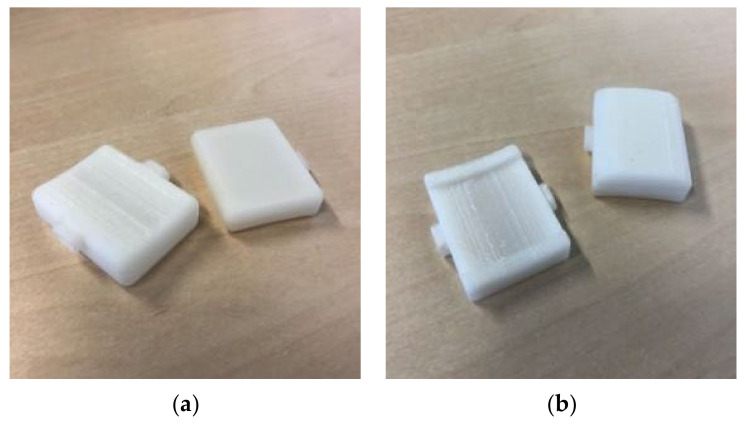
Modules: (**a**) flat body with flat lid; (**b**) rail body with curved lid.

The modules (Figure 4B; Figure 6) consist of two pieces: the body and the lid; these are manufactured by 3D printing in acrylonitrile butadiene styrene (ABS). The body is the element that keeps its measurements constant, while the lid varies in height depending on what the module contains. This allows different components to be accommodated just by changing one measure. In our case, the gap that houses the electronics always maintains its height (32 mm) and its width (22 mm), combining its depths between 5.4 mm for electronic modules and 16.4 mm for battery modules.

In terms of shape, two types of body (Figure 5B) were tested depending on the width of the strap: a flat body simply attached on the wide strap, and a rail body that embraces the narrow strap, generating battlements between module and module. To enhance the integration of the modules on the strap, the base of the body is provided with a curvature that accompanies the circumference of the animal’s neck.

As for the lids, a flat version and a more curved one (Figure 5B) are proposed. The flat lid reduces the material and thickness of the modules to the minimum, while the curved lid follows the curvature of the collar and generates fewer edges, although the thickness of the modules increases.

The union between the body and the lid is carried out by means of the adhesive and sealing of both parts by the chemical reaction that occurs between ABS and acetone in a tongue and groove that runs along its perimeter. Both body options have a ledge on each side where a heat shrink tube adheres and compresses. This allows, on one hand, for the protection of the connection of the modules that is made by wires, and on the other, to join the modules together. Modules were attached on the strap using double-sided tape.

#### 3.2.2. Electronics

Building blocks of the electronics inside the collar vary according to the target animal and the monitoring features required (Table 3).

**Table 3:** Electronic features depending on the type of monitoring.

	Intensive Monitoring	Remote Monitoring
Target animals	Small-medium sized mammals (sheep, goat, etc.)	Medium and large sized mammals (horse, cow, etc.)
Monitoring scenario	Animals are estabulated or in confined facilities that allow periodic check in.	Animals are free and move in large areas not seen for months.
Electronic blocks	1 block with: Movement and magnetic sensor SD card for massive sensor datalogging, Bluetooth for communication and proximity sensing (Figure 7).	3 blocks with: Movement sensor with smart analysis to extract activity. GPS (including antenna). Lora communication.
Battery	6000 mA·h Li-Ion battery made up by 6 pieces of 1 A·h	4000 mA·h Li-Ion battery made up by 4 pieces of 1 A·h
Energy expenditure	Low power (when no movement detected): 11.2 J/day Sensor datalogging (5’ proximity scan and 16 h of movement recorded): 663.5 J/day Data downloading (30’ once per day): 46.8 J/day	Low power (when no movement detected): 33.7 J/day Sensor data logging (1 h proximity scan and 2 h of movement recorded): 77.7 J/day GPS data logging (24 locations/day) and activity sending (1 h periodicity): 62.6 J/day GPS data logging (4 locations/day): 10.4 J/day
Device lifetime	91 days	Smart mode (24 gps/day) + Activity + BLE --> 236 days Smart mode (4 gps/day) --> 5.5 years

- Sensing, computing, and datalogging: these were implemented using a microcontroller (to manage data and rest of the hardware) and small sensors measuring linear and angular acceleration, sound, magnetic field, etc.
- Communications: these were implemented using different communication modules depending on the required range, data throughput, and antenna size (e.g., Bluetooth (short range, high throughput, smallest antenna), VHF (very long range, very little throughput, large antenna) and Lora (long range, low throughput, small antenna)).
- Location: this can be undertaken using a global navigation satellite system (GNSS) module for precise and global location or using wireless communication modules for rough positioning.
- Energy: battery is required to run the electronics and its technology and size defines the system’s lifetime by dividing the energy available inside the battery by the energy required by the electronics (calculated as the sum of the products of the power required by each electronic block inside the device times the time this piece is running).
  (1) $$\mathrm{lifetime}=\frac{\mathrm{battery}\_\mathrm{energy}\;}{\sum_{\mathrm{electronic}\_\mathrm{blocks}}\left(\mathrm{running}\_\mathrm{power}\;\times \;\mathrm{time}\_\mathrm{running}\right)}$$

We designed two different electronics that fit inside the collar; all of them fulfilled the dimensional restrictions of the maximum area of 20 mm × 30 mm.

### 3.3. Coating

The coating of the collar (Figure 4C) must have a double function, reinforce it against climatic conditions and resist the manipulation of the various elements that compose it. For this reason, conventional heat shrink tubing was used to cover the collar and its ends were sealed with adhesive heat shrink strips (Figure 5C). Conventional heat shrink was also used to hide mechanical joints that have shiny elements, in order not to attract the attention of the animal, its companions or other species.

### 3.4. Drop-Off

**Figure 8:**
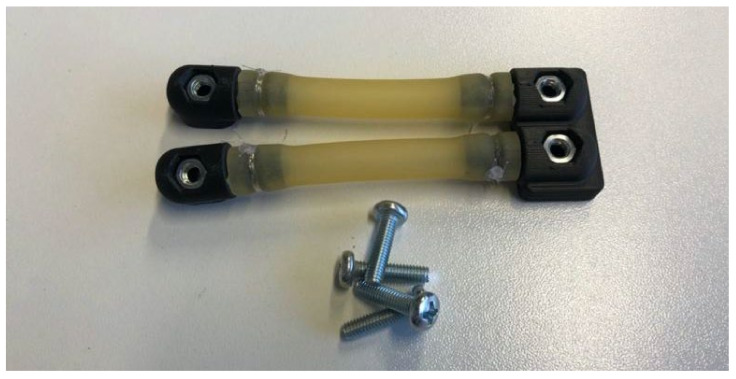
Drop-off system: two pieces are used at left and a single piece is used at right, in both cases standard robustness.

Drop-off systems, typical in the study of wildlife, can be used as a security system in case the animal is trapped because of the collar, which can be an interesting element to also incorporate in intelligent farming. The drop-off system (Figure 4D; Figure 8) that the collar had is similar to that of Telonics commercial solutions [46], since we considered it to be a successful method as a safety system against hanging. This was based on the degradation of latex against the action of the environment. This system consists of two latex tubes that are joined by means of nylon thread to the connecting ends of some pieces fixed to the strap thanks to a nut–screw connection. Nevertheless, certain novelties that improve the design of the commercial models on several levels have been introduced in regard to fasteners. The redesign joins the two elements of the commercial model in a single piece in such a way that its assembly is facilitated and its robustness is increased. Moreover, it offers the possibility of combining or choosing between the two structure options, single or double, in order to adapt the collar to each context and animal species (in some cases the double option could improve its resistance) (Figure 5D).

### 3.5. Closure

The closures that are currently used in market devices make the placing of the collar a complex and time-consuming activity. The most difficult issue that surrounds this element is that it must attend to the needs of two main types of user: animal (it must resist its force and have a mechanism that is difficult for them to open) and the veterinarian (it must be easy and quick to open for veterinarians).

**Figure 9:**
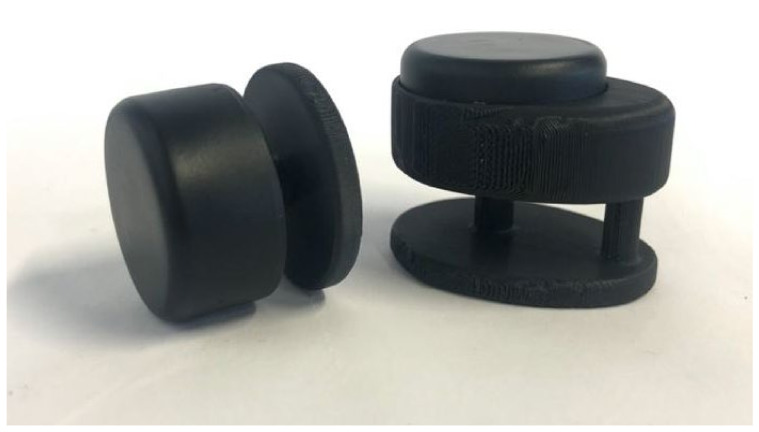
Magnetic closure: single pin closure (**left**) and double pin closure (**right**).

To improve this problem, we developed a closing system (Figure 4E; Figure 9) based on a magnetic head that locks and unlocks on a pin thanks to the action of a neodymium magnet. Two concepts were designed (Figure 5E): a version composed of (i) a base with two pins, a first pin that allows closure and a second pin that keeps the collar fixed without allowing it to rotate; and (ii) an upper piece that contains the magnetic mechanism and guides the second pin; and a second version with a single pin to allow us to learn whether the use of the second pin is really necessary or if, morphometrically, there is not enough clearance for the strap to rotate too much on the animal’s neck. This latest version has a top piece that contains the magnetic mechanism and a base with a single pin.

## 4. Discussion

**Figure 10:**
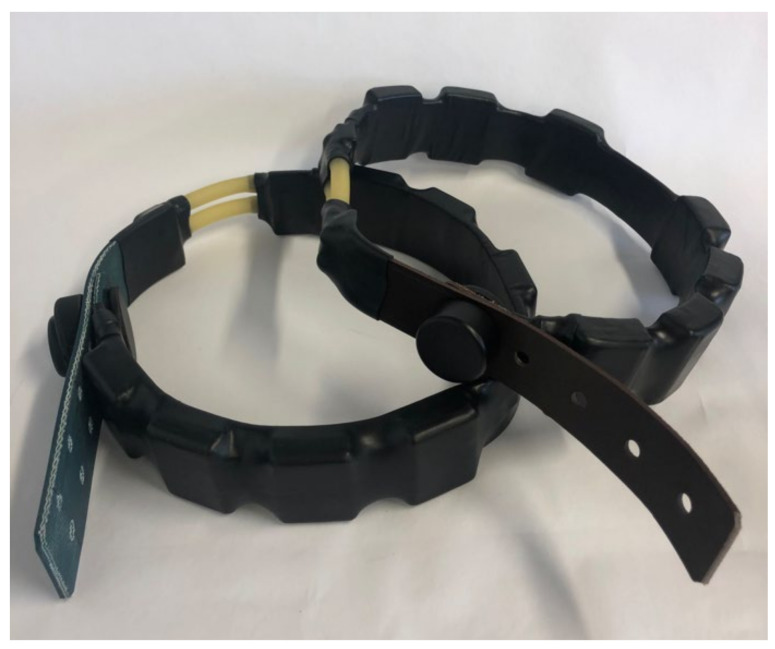
High fidelity prototypes developed to evaluate the range of options available. The elements that have more than one design proposal are represented in one or the other collar, being totally interchangeable.

The device presented aims to resolve several issues identified in the current animal telemetry offers: weight distribution, autonomy, flexibility in design, and price. The device evaluation was carried out using a mixed methods approach [43,44,45] using quantitative techniques such as laboratory experiments, current offer comparison tables, as well as qualitative (e.g., interviews, focus group, observations) following the Xassess design evaluation method [43]. This evaluation relies on a high number of prototyping iterations of local parts, but also of the entire product (Figure 10).

**Figure 11:**
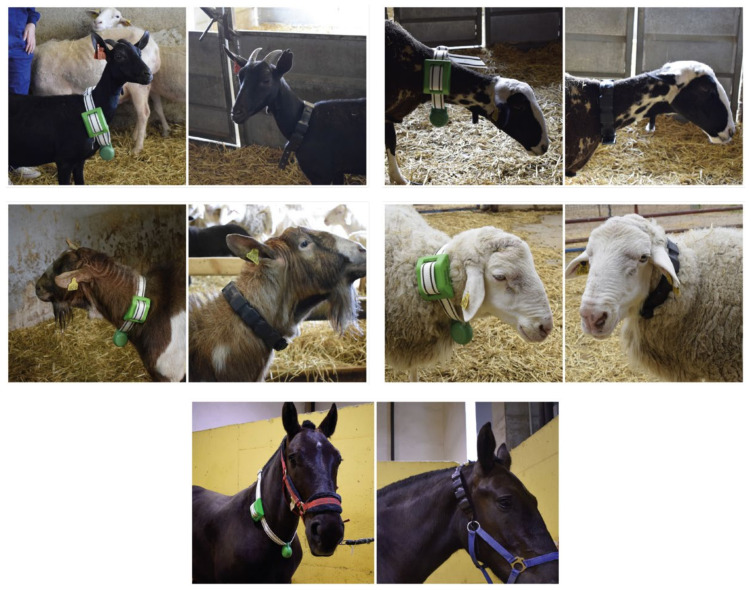
Different animal species with: a commercial collar [36] (left image of each of the pairs, green collar); and with the proposed collar (right image of each of the pairs, black collar).

Several experts from different institutions participated in the evaluation of the product: four veterinarians; three technical personnel who work in continuous contact with animals; four behavioral researchers; and two managers of animal centers. The collars were placed on sheep (rasa aragonesa and roya bilbilitana) and goats (murciano granadina and mestizo de Florida), as seen in (Figure 11), and they have also been placed as part of a horse (hispano-bretón) halter, demonstrating their adaptability.

### 4.1. Design Flexibility

The recent popularization of telemetry for animal research has led to the emergence of increasingly diverse projects with more specific requirements. This means that, on occasion, the market offers are not adapted to the needs of the project and alternative solutions have to be sought such as individual customization of the devices. Until recently, these modifications involved manufacturing processes whose costs made the project unviable [47], having a negative impact on the size of the animal samples studied [48] and limiting the variability of solutions adapted to different animals or to different technical requirements (batteries of different sizes depending on the needs, adapted communication modules, etc.).

The new concept of modular design of the wearable as well as the particular design of the modules that we propose highlight the fractioning of the electronics, which allow the product to be adapted to the project requirements by customizing the components, and therefore the functions, providing flexibility. This is possible thanks to the modular system of bodies and lids, which adapts the size of the module to the component it houses.

### 4.2. Weight and Weight Distribution: Comfort and Autonomy

The main weight of the wearables that are currently available on the market is concentrated in the telemetric device itself and this is generally placed at the bottom, so there is no homogeneous distribution of weight (Figure 11). Our proposal distributes the weight along the entire strap, distributing the electronic components in at least six modules (Figure 10). The homogeneous distribution of the elements of the collar also allows the thickness of the collar to be more homogeneous (Table 4). According to the experts, both results are translated into greater comfort for the animal.

The autonomy of the devices is another point of concern. The current autonomy is often not sufficient and therefore the animals cannot be monitored for the desired time [26]. The size and weight of the electronic device of the commercial collars is determined by the battery life; the larger the battery, the larger the electronic element and the heavier the collar. The weight distribution and the modular nature of the proposal show that, although the autonomy of the battery is increased, the resulting increase in weight and size can be distributed along the collar (Table 5).

### 4.3. Structure

The strap forms the main structure of the collar, therefore a correct definition of its material (leather or rubber–canvas) and width (the same width as the modules or a little less) is vitally important.

The best material for the strap is leather because it is lighter and adapts better to the movements of the animals and therefore is more comfortable. Rubber–canvas is stronger but leather is sufficient for farm animals: the neck is a protected part of the body and they are herbivorous animals with blunt teeth.

Regarding the width of the strap, a similar reasoning is followed: a narrower width than the modules is less bulky and provides movement to the collar; and therefore, increases comfort by being resistant enough for use with farm animals.

### 4.4. Unions

The union between the different elements of the design is usually made by rivets and/or nut–screw unions (Table 6), mechanical elements that increase the number of parts of the product and its weight. Our modules are attached on the strap and it is the coating that finishes fixing them. This registration is carried out using double-sided tape, which translates into extra-light joints and with a reduced number of pieces (one piece per module compared to 6–8 on the market).

### 4.5. Closing System

The closing of the devices is done mechanically with threaded connections, and is one of the most critical points in the sequence of use as it takes too long (Table 7). However, the proposed closure system allows much faster manipulation of the collar. In addition, it reduces the number of parts of the closure and standardizes the opening tool: a neodymium magnet.

Regarding the structure of the closures, it is considered that both versions are easy to understand and use and that the force to be applied in the system is correct. However, the double pin version prevents the strap from rotating on itself unlike the single pin version. This limits the movements of the collar and makes the data from the inertial sensors more accurate.

### 4.6. Integration of the Elements

On many occasions, the elements that make up the wearable are not formally integrated (Table 8), and it is believed that animals that use wearables stand out more among predators [26]. We propose a device that formally adapts to the context and that integrates the elements, thanks to several design decisions: (i) homogeneous distribution of the weights, which favors that the visual mass of the collar is distributed throughout it; (ii) maintain a similar thickness throughout the entire collar; (iii) apply heat shrink tubing as a coating over most of the collar to homogenize the device; (iv) hide shiny elements; (v) use an internal antenna to avoid manipulative elements outside; and (vi) use rounded and smooth shapes that adapt to the morphometry of the animal.

## 5. Conclusions

Animal telemetry is a topic with a great future within intelligent animal farming, but where serious design-dependent problems are evident. The objective of the project was to cover the niches that have been deduced from the study of scientific literature and the market and to provide solutions from the application of design.

The presented device represents a telemetric option whose design process has put the user at the center, especially the animal user, through an animal-centered design strategy that could be followed in future research. In this way, the concept of the GPS collar has evolved, traditionally chaired by a central module that housed practically all the electronic elements and that did not attend to the premise that wearable devices must be able to collect accurate and reliable data without influencing the behaviors and activities of carrier users [49]. This device solves many of the existing problems in animal telemetry devices and contributes to improving the current offer on the market:

- Homogeneous distribution of weight in at least six modules;
- Three times lighter than devices on the market with the highest number of modules (2–3 modules);
- Design flexibility: modularity and 3D printing;
- Modular electronics on demand of the project with customizable functions;
- Extra-light unions with a reduced number of pieces (one piece per module compared to 6–8 on the market);
- Tightness and resistance to environmental conditions;
- Collar thickness of at least 50% less than that of commercial devices;
- Quick magnetic closure system;
- Wirelessly rechargeable batteries and homogeneous distribution on the collar in case of higher demand; and
- Formal adaptation to the requirements of the context and visually integrated elements.

The results of this research are of interest to designers and manufacturers of animal telemetry, technologists, and professionals in the animal and farm sector, since they contribute to the knowledge about animal monitoring through the design of the device itself and the methodological approach used for its achievement.

## Figures and Tables

**Figure 7:**
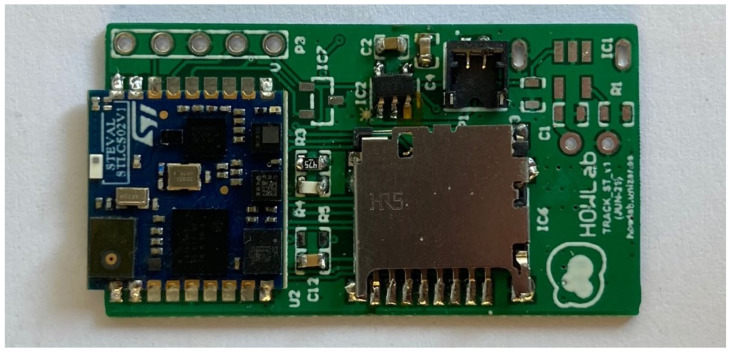
Electronic block for intensive monitoring (20 × 30 mm).

**Table 1:** Design strategy followed for the development of Phase 1.

Objective	Analysis & Source	Method
Obtain information from the telemetric context in which the project will be developed; and compile recommendations and solutions resulting from the investigative exercise.	State of the art from papers in scientific journals.	Literature review.
Extract considerations to take into account in relation to environmental conditions and use cases.	Analysis of the environment of use through semi-structured interviews with users.	Synthesis of the information according to cases of use and location of the animals.
Define user profiles; and detect their needs.	User analysis through semi-structured interviews with users.	Person method (modeling the characteristics of the different groups of users). Quotes (collect literary phrases that express the wishes or concerns of users). Team meetings to synthesize the information.
Define the morphometric measurements of the animals that determine the design of the device; and decide what type of device is going to be developed according to its placement.	Morphometric analysis by semi-structured interviews with users and morphometric tables.	Information synthesis.
Know what is currently being offered in terms of telemetric devices and what market niches or problems currently exist in it.	Market study and structural analysis from market offer and scientific papers.	Search for products by manufacturers. Synthesis of the characteristics belonging to the elements that make up a standard collar.
Decide what functions are going to be implemented; and define the electronic components that the wearable must have.	Functional analysis through meetings with users and with the team.	Information synthesis.
Define a manufacturing strategy that reduces production costs.	Manufacturing Context studied from papers in scientific journals.	Literature review.

**Table 2:** Objectives evaluated in relation to the sources and the methods used for their evaluation.

	Objectives to Evaluate		Source ^1^	Methods ^2^
Elements	Strap	Material (malleability; and resistance to environmental conditions)	EE	IM + RLT + SSI
		Width (adaptation to the morphometry of the animal)	EE	IM + RLT + SSI
		Length (adaptation to the morphometry of the animal)	EE	IM + RLT + SSI
	Modules—Body	Shape (comfort for the animal)	EE + E	IM + RLT + SSI + LE
	Modules—Lid	Shape (comfort for the animal)	EE + E	IM + RLT + SSI + LE
	Coating	Adaptation to the collar	E	LE
		Resistance (to be worn)	EE	IM + RLT + SSI
	Drop-Off	Robustness	EE + E	IM + RLT + SSI + LE
		Structure (change proposed)	EE + E	IM + RLT + SSI + LE
	Closure	Structure (new design)	EE + E	IM + RLT + SSI + LE
		Ease of use	EE + RE + E	IM + FG + RLT + SSI + LE
		Force applied (required for handling)	EE	IM + RLT + SSI
	Unions	Weight reduction	CO	DA
		Body—Lid sealing	E	LE
		Module—Module sealing	E	LE
Composition	Collar	Weight	EE + RE + CO	RLT + SSI + DA + IM
		Weight distribution	EE + RE + CO + E	RLT + SSI + DA + IM + FG + LE
		Integration of elements and formal and aesthetic adaptation	EE + RE + CO	RLT + SSI + DA + IM + FG
		Autonomy	CO	DA
		Design flexibility	CO	DA
		Comfort for the animal	EE + RE	RLT + SSI + IM + FG
		Ease of use	EE + RE + CO	RLT + SSI + DA + IM + FG
		Interaction	EE + RE	RLT + SSI + IM + FG

^1^ Source abbreviations: EE (Experts in the Environment); E (Engineers); RE (Research Experts); CO (Current Offer). ^2^ Methods abbreviations: IM (Iterative Methods with experts); RLT (Real-Life Testing); SSI (Semi-Structured Interviews); LE (Laboratory Experiments); FG (Focus Group); DA (Document Analysis).

**Table 4:** Current offer comparison table: weight and weight distribution.

Device	Weight	Weight Distribution (L × W × H mm^3^)
Our Proposal	210 g (Collar A) 270 g (Collar B)	7 modules: 4 large modules of (30.4 × 40.4 × 11) and 3 small modules of (30.4 × 40.4 × 8)
Personalized Telonics Collar	238 g	1 module (Approx. 55 × 38 × 28)
Telonics, TGW-4570-4	500–880 g	3 modules (73 × 51 × 37)
Telemetry Solutions, Iridium GPS Collar	125–250 g	2 modules (-)
Tellus, Small Personalizable	>600 g	2 modules (76 × 56 × 55)
Advanced Telemetry Systems, G2110E2 Iridium	825 g	2 modules (115 × 80 × 65)
Advanced Telemetry Systems, G5-D Iridium	500 g	2 modules (70 × 50 × 47)
Lotek, Ultimate V6C 176G	278–325 g	1 module (88 × 32 × 30)
Lotek, WILDCELL MG	950 g	2 modules (120 × 86 × 126)
Lotek, PinnaclePro L	630–670 g	3 modules (-)
Ixorigue, GPS Ixotrack	960 g	1 module (83 × 113 × 38)
Open-source collar for terrestrial animals over 8 kg [19]	240 gr	1 module (62 × 38 × 32)

**Table 5:** Current offer comparison table: modules on which autonomy depends and operational life.

Device	Autonomy Dependent On	Operational Life
Our Proposal	(at least) 3 modules	Intensive monitoring Smart mode + Activity + Bluetooth Low Energy (BLE) + Sending data—91 days
		Remote monitoring Smart mode (24 gps/day) + Activity + BLE—236 days Smart mode (4 gps/day)—2021 days
Personalized Telonics Collar	1 module	-
Telonics, TGW-4570-4	1 module	4 gps/day, No Very High Frequency (VHF)—6.2 years 4 gps/day, VHF 4 h/day—5.1 years
Telemetry Solutions, Iridium GPS Collar	1 module	-
Tellus, Small Personalizable	1 module	-
Advanced Telemetry Systems, G2110E2 Iridium	1 module	VHF on 8 h/day, 12 locations/day—3 years VHF on 8 h/day, 3 locations/day—4 years
Advanced Telemetry Systems, G5-D Iridium	1 module	VHF on 8 h/day, 6 locations/day, uplinked every 2 days—4 years
Lotek, Ultimate V6C 176G	1 module	60 ppm VHF—776 days
Lotek, WILDCELL MG	1 module	50 min between gps fixes. An SMS message is sent after 7 acquired gps fixes—2 years
Lotek, PinnaclePro L	1 module	VHF beacon is set to operate for 1 h a day at the average. The collar transmits through Iridium after collecting 18 positions, 7 positions/day—4 years
Ixorigue, GPS Ixotrack	1 module	24 gps/day—1 year
Open-source collar for terrestrial animals over 8 kg [19]	1 module	24 gps/day—103 days

**Table 6:** Current offer comparison table: union weight.

Device	Unions Weight (g) ^1^
Our Proposal	1.05 g (0.15 × 7 pieces)—7 pieces of double-sided tape
Personalized Telonics Collar	1.2 g (0.15 × 8 pieces)—4 double-sided rivets
Telonics, TGW-4570-4	3.6 g (0.15 × 24 pieces)—12 double-sided rivets
Telemetry Solutions, Iridium GPS Collar	-
Tellus, Small Personalizable	30.4 g (7.6 × 4)—16 pieces, 4 base sets with rods, plate and 2 self-locking nuts
Advanced Telemetry Systems, G2110E2 Iridium	22.8 g (7.6 × 3)—12 pieces, 4 base sets with rods, plate and 2 self-locking nuts
Advanced Telemetry Systems, G5-D Iridium	15.6 g (2.6 × 6) 12 pieces, 6 sets of screw + self-locking nut
Lotek, Ultimate V6C 176G	-
Lotek, WILDCELL MG	15.2 g (7.6 × 2)—8 pieces, 4 base sets with rods, plate and 2 self-locking nuts
Lotek, PinnaclePro L	31.2 g (2.6 × 12) 24 pieces, 12 sets of screw + self-locking nut
Ixorigue, GPS Ixotrack	It does not use mechanical unions to fix the module to the strap, however, it does use a shot on the bottom part of the collar (500 g) to keep it in the correct position.
Open-source collar for terrestrial animals over 8 kg [19]	10.4 g (2.6 × 4) 8 pieces, 4 sets of screw + self-locking nut

^1^ The weight of the unions in commercial devices has been estimated from the weights of various commercial mechanical elements. Each piece of a rivet is considered to weigh approximately 0.15 g; each base set with rods, plate, and 2 self-locking nuts is considered to weigh approximately 7.6 grams; each set of screw + self-locking nut is considered to weigh approximately 2.6 grams. The rest of the numerical data were extracted from the characteristics specified by the manufacturers of each device.

**Table 7:** Current offer comparison table: ease of use.

Device	Ease of Use
Our Proposal	Magnetic closure
Personalized Telonics Collar	Mechanical nut–screw closure
Telonics, TGW-4570-4	Mechanical nut–screw closure
Telemetry Solutions, Iridium GPS Collar	Mechanical nut–screw closure
Tellus, Small Personalizable	Mechanical nut–screw closure
Advanced Telemetry Systems, G2110E2 Iridium	Mechanical nut–screw closure
Advanced Telemetry Systems, G5-D Iridium	Mechanical nut–screw closure
Lotek, Ultimate V6C 176G	Mechanical nut–screw closure
Lotek, WILDCELL MG	Mechanical nut–screw closure
Lotek, PinnaclePro L	Mechanical nut–screw closure
Ixorigue, GPS Ixotrack	Metal buckle.
Open-source collar for terrestrial animals over 8 kg [19]	Metal buckle.

**Table 8:** Current offer comparison table: integration of elements and formal and aesthetic adaptation.

Device	Integration of Elements and Formal and Aesthetic Adaptation
Our Proposal	Elements with similar thickness (8 mm the minimum and 20.5 mm maximum and the maximum is between a piece of 19 mm and another of 13 mm) distributed along the collar. A single coating. Antenna integrated in PCB, without external elements. Smooth and rounded finishes. Curvature in the body of the module that adapts to the neck of the animal. Hidden shiny elements.
Personalized Telonics Collar	Large main element at the bottom (28 mm). Heat shrinkable in the Drop-Off area. External antenna. Edges at the top and bottom, although rounded at the front. Curvature in the body of the module that adapts to the neck of the animal. Hidden glossy elements except for the closure.
Telonics, TGW-4570-4	Large main element at the bottom (37 mm). It does not use heat shrink, the coating is sandwich type. Internal antenna. Slightly rounded edges. Curvature in the body of the module that adapts to the neck of the animal. Bright elements exposed.
Telemetry Solutions, Iridium GPS Collar	Great main element at the bottom. Heat shrinkable only on modules. Internal antenna. Modules with irregular shapes. No curvature in the body of the module to adapt to the neck of the animal. Bright elements exposed.
Tellus, Small Personalizable	Large main element at the bottom (55 mm). Without cover. Internal antenna. Modules with slightly rounded edges. No curvature in the body of the module to adapt to the neck of the animal. Bright elements exposed.
Advanced Telemetry Systems, G2110E2 Iridium	Large main element at the bottom (65 mm). Without cover. External antenna. Modules with slightly rounded shapes and edges. With curvature in the body of the module to adapt to the neck of the animal. Bright elements exposed.
Advanced Telemetry Systems, G5-D Iridium	Two large main elements at the bottom (47 mm). Without cover. External antenna. Modules with slightly rounded shapes and edges. With curvature in the body of the module to adapt to the neck of the animal. Bright elements exposed.
Lotek, Ultimate V6C 176G	Large main element at the bottom (30 mm). Heat shrinkable coatings in specific locations. External antenna. Modules with slightly rounded edges. With curvature in the body of the module to adapt to the neck of the animal. Hidden glossy elements except for the closure.
Lotek, WILDCELL MG	Large main element at the bottom (126 mm). Without cover. Internal antenna. Module with robust and slightly rounded shapes, lid-body closure not visually integrated. With curvature in the body of the module to adapt to the neck of the animal. Bright elements exposed.
Lotek, PinnaclePro L	Great main element at the bottom. External antenna. Module with robust shapes and sharp edges. With curvature in the body of the module to adapt to the neck of the animal. Bright elements exposed.
Ixorigue, GPS Ixotrack	Large main element at the right side (38 mm). Without cover. Internal antenna. Module with robust and slightly rounded shapes, lid-body closure not visually integrated. No curvature in the body of the module to adapt to the neck of the animal. No shiny elements exposed except the closure.
Open-source collar for terrestrial animals over 8 kg [19]	Large main element at the bottom (32 mm). Without cover. Internal antenna. Edged module. With curvature in the body of the module to adapt to the neck of the animal. Bright elements exposed.

## Data Availability

Not applicable.
